# Factors associated with insecticide-treated net usage among women of childbearing age in Malawi: a multilevel analysis

**DOI:** 10.1186/s12936-018-2522-z

**Published:** 2018-10-19

**Authors:** Owen Nkoka, Ting-Wu Chuang, Kun-Yang Chuang, Yi-Hua Chen

**Affiliations:** 1 School of Public Health, College of Public Health, Taipei Medical University, 250 Wu-Hsing St., Taipei, 110 Taiwan; 2 Department of Molecular Parasitology and Tropical Diseases, School of Medicine, College of Medicine, Taipei Medical University, Taipei, Taiwan

**Keywords:** Insecticide-treated nets, Women of childbearing age, Malaria, Multilevel, Malawi

## Abstract

**Background:**

This study aimed to identify factors at individual and community level influencing insecticide-treated net (ITN) usage among groups of women of childbearing age (WOCBA) in Malawi.

**Methods:**

Factors influencing ITN usage in Malawi were assessed through interviews with 16,130 WOCBA (15–49 years) across 850 communities who participated in the 2015–2016 Malawi Demographic Health Survey. Multilevel logistic regression analysis was used.

**Results:**

ITN use was similar between pregnant women and non-pregnant women with children under 5 years (45.9% and 46.9%, respectively), but slightly lower among non-pregnant women without children under 5 years (39.1%). Both individual and community characteristics were associated with ITN use among WOCBA and varied significantly across subgroups. Specifically, non-pregnant women with children under 5 years living in communities where women had high autonomy in health care decisions had an 18% greater odds of using an ITN compared with those from communities where women had low health care autonomy (adjusted odds ratio [aOR] = 1.18; 95% confidence interval [CI] 1.00–1.38). Distance to health care facility influenced ITN usage among pregnant women; those who did not regard distance as a problem had a 44% greater odds of using an ITN than those for whom distance was seen as a problem (aOR = 1.44; 95% CI 1.09–1.89). Number of household members, region, urbanization, and community ITN coverage influenced ITN usage across all WOCBA groups.

**Conclusion:**

The findings confirmed the importance of assessing various factors affecting ITN usage among groups of WOCBA. Both individual- and community-level factors should be considered when designing and implementing ITN programmes in Malawi.

## Background

Use of insecticide-treated nets (ITNs) is one of the core vector control methods for malaria prevention and has been shown to reduce malaria incidence by 50% in several malaria-endemic countries [1]. The effects of malaria are especially strong among pregnant women, for whom malaria may cause maternal anaemia, preterm delivery, and low birthweight [2–4]. High ITN coverage in sub-Saharan Africa has been reported; however, discrepancies in ITN use in the region remain problematic [5–7].

In Malawi, ITNs are at the centre of malaria-control initiatives. A nationwide mass ITN-distribution campaign took place in 2012 [8]. The Malawi ITN policy recommends that free ITNs be given to women and children during antenatal care (ANC) and expanded programme on immunization (EPI) visits [8]. These efforts led to a rise in ITN use among pregnant women from 35% in 2010 to 62% in 2014 [9, 10]. However, a 2015 Malawian study revealed a drop in ITN use to 53% among women of childbearing age (WOCBA) who had visited ANC facilities [11]. This drop is a concern in the fight to eliminate malaria by 2030 [12].

A large body of research has demonstrated that factors such as women’s age [13], parity [14], education status [14], employment status [13, 15], household wealth [13, 16], and religion [13] have significant effects on ITN utilization. For instance, in Kenya, women who had received a higher level of education were twice as likely to use ITNs than women with no formal education [16]. In Cameroon, 45% of multiparous women reported having slept under an ITN the night before the survey, as compared with 21% of primigravida women [17]. However, inconsistent results have been obtained, with some studies indicating that age [14], parity [15], and education [18] have no significant associations with ITN use among pregnant women. The discrepancies in these findings may be accounted for by community characteristics, which have been shown to exert a strong influence on health outcomes and health care utilization across Africa [19, 20]. Indeed, community factors influence individual risk exposure and resource access. In Rwanda, community factors such as wealth were shown to influence ITN use among those aged under 5 years [21]. However, little is known about the effects of community on ITN use among women.

The health behaviours of WOCBA, including their adoption of malaria-prevention strategies, may be affected by their maternal status (i.e., pregnant, non-pregnant, with or without children aged under 5 years) [22, 23]. In addition, the relationships between ITN use and other factors such as women’s autonomy in health care decisions and women’s health behaviours have not been investigated. These factors among WOCBA groups, along with both individual- and community-level factors, should be investigated to ensure that future interventions, such as mass campaigns and health education messages, can be designed to reach vulnerable communities and groups of women.

Therefore, this study used a nationally representative data sample to investigate the factors at individual and community level associated with ITN use among WOCBA in Malawi. The factors affecting ITN usage among WOCBA groups (i.e., pregnant women and non-pregnant women with and without children aged under 5 years) were also analysed.

## Methods

### Study area and design

Malawi, located in Southern-Central Africa, experiences malaria transmission peaks between October and April [11]. This is a population-based cross-sectional study utilizing the Malawi Demographic and Health Survey (MDHS) data for 2015–2016.

### Survey and participants

Details regarding the scope and methodology of the MDHS have been published elsewhere [24]. Briefly, nationally representative samples were produced using a two-stage stratified cluster design. The first stage involved selection of clusters and household listings. The second stage involved household selection through the use of equal probability systematic criteria. A total of 30 and 33 households from urban and rural clusters, respectively, were selected. Specifically, the probability of households in each cluster was calculated by a simple formula: *p *=* n*/*N*, where p is the probability for each household in a cluster to be selected, *n* is the number of households selected in a cluster (30 or 33 per urban and rural cluster, respectively) and *N* is the population size (i.e. the total number of households listed in the household listing operation in a particular cluster) [24]. Thus, with the use of equal probability systematic criteria, each household in a cluster had a known and equal probability of being selected.

A total of 24,562 WOCBA were interviewed in the 2015–2016 MDHS. For this study, only married WOCBA (i.e., aged 15–49, n = 16,130) were included. Married women were selected because of the importance of women’s role in promoting health within families. Additionally, the question in the survey on decision making in health care had only been answered by married women; non-married women were not asked this question. There were a total of 8482 unmarried WOCBA in the MDHS (representing 34.33%). Among the unmarried women, those with children were 4574. Sensitivity analyses to include these unmarried women in the assessment of the distributions of key variables according to ITN usage were performed and the results were fairly consistent.

### Data collection

Data were collected through face-to-face interviews using pretested questionnaires. Information on sociodemographics, health-related factors, and malaria-prevention practices (such as ITN use) was collected through verbal reports. A total of 24,562 out of 25,146 eligible women were interviewed, representing a 98% response rate.

### Outcome variable: ITN use

An ITN was defined as a factory-treated net that did not require any further treatment (long-lasting ITN) or a net that had been soaked with insecticide within the past 12 months. ITN use was defined as sleeping under an ITN the night before the survey questions were answered (yes/no) [25].

### Independent variables

The study examined independent variables at two levels; level 1 (hereinafter referred to as individual-level factors) included participants’ responses to survey questions on the personal factors, and characteristics of household from which the individuals came from, while level 2 included community-level factors which were derived/aggregated from the individual-level factors [26].

#### Individual-level variables

The individual-level variables investigated included the sociodemographic factors age (15–24, 25–34, or ≥ 35 years), education (no education, primary, secondary, or above), occupation (employed or unemployed), and religion (Catholic, Protestant, Muslim, or other). Additionally, a wealth index was derived from measurements of household ownership (e.g., bicycle, radio). The weighted scores were categorized into three levels (poor, middle, or rich). Region of residence was expressed as northern, central, or southern, and residence was defined as urban or rural. Altitude was categorized as < 1600 or ≥ 1600 m [27]. Parity was categorized as primigravida, secundigravida, or multigravida. Number of children under 5 years was categorized as none, one, or ≥ 2, and number of household members as < 5 or ≥ 5 [24]. Individual health-related factors were also considered and included the number of ITNs in the household (< 2, ≥ 2), and indoor residual spraying (IRS) within the past 12 months (yes/no). Media exposure was measured by access to newspapers, radio, and television. Access to media was defined as those participants who reported to listening to radio at least once a week, watching television at least once a week, and reading newspaper at least once a week [24]. Survey respondents who had access to any of the three were considered to have media exposure. Distance to health facility was assessed by asking whether women perceived the distance to their nearest health facility as a problem, and women’s autonomy to make health care decisions was categorized as self, husband alone, and with others. Finally, water treatment and handwashing facility availability were assessed, and women with both, one, and neither were categorized as having good, medium, and poor health behaviours, respectively.

#### Community-level variables

To examine community-level factors, three community sociodemographic data points and programme community health factors were aggregated from the individual-level variables. Specifically, community-level employment, wealth, and education status were obtained by aggregating individual-level employment, wealth, and education status, respectively. The prevalence of employed and rich individuals with any education within each community was calculated. The resultant proportions were divided into three levels (tertiles): low, middle, and high. Similarly, community-level health factors were calculated using the proportion of women who did not perceive the distance to their nearest health facility as a problem (community-level distance to health facility), those from households with ≥ 2 ITNs (community-level ITN coverage), those with autonomy in health care decisions (community-level women’s autonomy in health care decisions), and those from households where insecticide had been sprayed within the past 12 months (community-level IRS coverage). The community-level health factor results were categorized as low, middle, or high.

### Ethics statement

The survey protocol was reviewed and approved by the National Health Sciences Research Board of Malawi, Institutional Review Board (IRB) of ICF Macro, and Centers for Disease Control (CDC) in Atlanta. Informed consent was obtained at the beginning of each interview, and the Demographic and Health Survey Programme granted permission for data analysis.

### Statistical analyses

The distributions of participants’ characteristics according to ITN use were examined using Chi square tests. In addition, factors associated with ITN use may differ among the WOCBA groups of pregnant women and non-pregnant women with or without under-5-year-old children (expressed as non-pregnant women with children under 5 years and non-pregnant women without children under 5 years, respectively); thus, the potential modifying effects of the three groups were examined. Interaction terms had *p*-values < 0.1; therefore, stratified analyses for the three groups were performed, and the results are presented separately. The pregnant women were not stratified according to having or not having children under 5 years because the sample sizes within groups upon stratification were small (i.e. ranged from 1 to 5 observations) to possibly bias the efficiency of multilevel random parameter estimates downwards [28–30]. Therefore, the pregnant women, being a vulnerable population by themselves, were examined regardless of their having children under 5 years or not.

### Modelling approaches

A two-level multilevel logistic regression analysis was applied to assess the effects of individual- and community-level factors on ITN use, for which women (level 1) were clustered within their communities (level 2). The associations (fixed effects) were reported as odds ratios (ORs) with corresponding 95% confidence intervals (CIs). Programme models were fitted. A null model with no explanatory variables was constructed to assess total variance between communities. Models I and II included individual- and community-level factors for estimation, respectively, and finally, Model III accounted for all individual- and community-level factors.

#### Measures of variation (random effects)

Variation was examined through area variance with corresponding 95% CI, the intraclass correlation coefficient (ICC), the median odds ratio (MOR), and the proportional change in variance (PCV) [31, 32].

#### Model fit testing

The Akaike information criterion (AIC) was used to test the goodness-of-fit of each model, with a lower value representing a closer model fit. To determine whether multicollinearity existed, the variance inflation factor (VIF) was used [33]. None of the variables displayed multicollinearity problems (all VIF < 10). All analyses were performed using Stata version 15.0 (Stata Corp LP, College Station, TX, USA).

## Results

The study surveyed 16,130 married women nested within 850 communities. Approximately 10.1% (1634) of the women were pregnant, 69.4% (11,197) were non-pregnant with children under 5 years, and 20.5% (3299) were non-pregnant without children under 5 years. The groups of women were mutually exclusive; women who were pregnant were placed in the pregnant women group regardless of whether they had any children under 5 years. ITN use among the three groups was approximately 45.9%, 46.9%, and 39.1%, respectively.

### Distribution of participants according to ITN use

The distribution of individual- and community-level factors according to ITN use were calculated and are presented in Table 1. In all study groups, significant differences (*p *< 0.05) were determined between ITN users and nonusers for six individual-level factors (i.e., education, wealth, number of household members, number of ITNs in household, media exposure, and distance to health facility) and based on community ITN coverage. Differences were found for the other factors among the three WOCBA groups.

**Table 1:** Distribution of participants according to ITN use

Variable	Pregnant women n = 1634			Nonpregnant women with children under 5 years n = 11,197			Nonpregnant women without children under 5 years n = 3299		
	n (%) No n = 883	n (%) Yes n = 751	*p*-value^a^	n (%) No n = 5951	n (%) Yes n = 5246	*p*-value^a^	n (%) No n = 2009	n (%) Yes n = 1290	*p*-value^a^
Individual-level factors									
Age (years)			0.977			< 0.001***			0.061
15–24	482 (54.3)	405 (45.7)		1799 (51.3)	1709 (48.7)		321 (65.1)	172 (34.9)	
25–34	308 (53.7)	266 (46.2)		2538 (52.2)	2326 (47.8)		487 (63.5)	280 (36.5)	
≥ 35	93 (53.8)	80 (46.2)		1614 (57.1)	1211 (42.9)		1201 (58.9)	838 (41.1)	
Education			0.001**			< 0.001***			< 0.001***
No education	100 (17.4)	475 (82.6)		952 (62.4)	573 (37.6)		405 (66.1)	208 (33.9)	
Primary education	599 (55.7)	476 (44.3)		4046 (54.9)	3316 (45.1)		1219 (63.1)	713 (36.9)	
Secondary+	184 (42.2)	252 (57.8)		953 (41.3)	1357 (58.7)		385 (51.1)	369 (48.9)	
Occupation			0.643			0.064			0.038*
Unemployed	291 (55.0)	238 (45.0)		1656 (51.3)	1574 (46.7)		494 (64.8)	268 (35.2)	
Employed	592 (53.6)	513 (46.4)		4295 (53.9)	3672 (46.1)		1515 (59.7)	1022 (40.3)	
Religion			0.925			0.073			0.729
Catholic	158 (50.8)	153 (49.2)		1067 (50.4)	1051 (49.6)		438 (60.2)	290 (39.8)	
Protestant	569 (54.3)	478 (45.7)		4000 (54.9)	3280 (45.1)		1370 (61.4)	860 (38.6)	
Muslim and other	156 (56.5)	120 (43.5)		884 (55.3)	715 (44.7)		201 (58.9)	140 (41.1)	
Wealth			0.005**			< 0.001***			< 0.001***
Poor	449 (59.1)	311 (40.9)		2782 (59.2)	1916 (40.8)		627 (67.8)	298 (32.2)	
Middle	153 (51.0)	147 (49.0)		1190 (52.8)	1064 (47.2)		402 (63.2)	234 (36.8)	
Rich	281 (49.0)	293 (51.0)		1979 (46.6)	2266 (53.4)		980 (56.4)	758 (43.6)	
Region			0.487			0.579			0.873
Northern	117 (54.9)	96 (45.1)		731 (51.6)	686 (48.4)		231 (62.4)	139 (37.6)	
Central	399 (54.4)	335 (45.6)		2569 (53.9)	2195 (46.1)		888 (60.1)	589 (39.9)	
Southern	367 (54.3)	320 (46.6)		2651 (52.9)	2365 (47.1)		890 (60.9)	572 (39.1)	
Residence			0.056			< 0.001***			0.016*
Rural	778 (55.4)	626 (44.6)		5195 (54.6)	4325 (45.4)		1628 (65.2)	867 (34.8)	
Urban	105 (45.7)	125 (54.3)		756 (45.1)	920 (54.9)		381 (54.1)	323 (45.9)	
Altitude (m)			0.863			0.922			0.168
< 1600	876 (54.0)	745 (46.0)		5916 (53.1)	5215 (46.9)		1994 (60.8)	1285 (39.2)	
≥ 1600	7 (53.8)	6 (46.2)		35 (45.0)	31 (47.0)		15 (75.0)	5 (15.0)	
Parity			< 0.001***			< 0.001***			0.189
Primigravida	275 (62.8)	163 (37.2)		61 (81.3)	14 (18.7)		282 (66.5)	142 (33.5)	
Secundigravida	168 (43.5)	218 (56.5)		1011 (49.6)	1027 (50.4)		193 (58.5)	137 (41.5)	
Multigravida	440 (54.3)	370 (45.7)		4879 (53.7)	4205 (46.3)		1535 (60.3)	1011 (39.7)	
Children under 5 years in HH			0.682			0.739			–
None	350 (55.5)	281 (44.5)		NA	NA		NA	NA	
One	410 (53.5)	356 (46.5)		3602 (53.3)	3155 (46.7)		NA	NA	
≥ 2	123 (52.0)	114 (48.0)		2349 (52.9)	2091 47.1)		NA	NA	
Number of HH members			0.011*			< 0.001***			0.049*
< 5	505 (50.8)	489 (49.2)		1888 (47.7)	2072 (52.3)		943 (58.5)	670 (41.5)	
≥ 5	378 (59.1)	262 (40.9)		4063 (56.1)	3174 (43.9)		1066 (63.2)	620 (36.8)	
Number of ITNs in HH			< 0.001***			< 0.001***			< 0.001***
< 2	730 (64.8)	397 (35.2)		4835 (68.7)	2202 (31.3)		1689 (80.0)	423 (20.0)	
≥ 2	153 (30.2)	354 (69.8)		1116 (26.8)	3044 (73.2)		320 (27.0)	867 (73.0)	
IRS			0.234			0.340			0.234
No	832 (54.6)	693 (45.4)		5605 (53.3)	4909 (46.7)		1918 (61.2)	1215 (38.8)	
Yes	51 (46.8)	58 (53.2)		346 (50.7)	336 (49.3)		91 (54.8)	75 (45.2)	
Media exposure			0.013*			< 0.001***			< 0.001***
No	582 (57.2)	435 (42.8)		3946 (56.3)	3063 (43.7)		1241 (64.4)	685 (35.6)	
Yes	301 (48.8)	316 (51.2)		2005 (47.9)	2183 (52.1)		768 (55.9)	605 (44.1)	
Distance to HF			0.001**			0.001**			<0.001**
No problem	330 (48.2)	354 (51.8)		2462 (50.7)	2392 (49.3)		856 (56.8)	651 (43.2)	
Problem	553 (58.2)	397 (41.8)		3489 (55.0)	2854 (45.0)		1153 (64.3)	639 (35.7)	
Women’s autonomy in HC decisions			0.791			0.088			0.468
Self	133 (55.6)	106 (44.4)		1160 (55.1)	944 (44.9)		405 (61.4)	255 (38.6)	
Husband alone	305 (52.6)	275 (47.4)		1929 (54.0)	1641 (46.0)		600 (62.8)	355 (37.2)	
With others	445 (54.6)	370 (45.4)		2862 (51.8)	2661 (48.2)		1005 (59.6)	680 (40.4)	
Health behaviour			0.529			< 0.001***			0.008**
Poor	115 (55.3)	93 (44.7)		788 (60.6)	512 (39.4)		225 (68.2)	105 (31.8)	
Moderate	441 (49.6)	448 (50.4)		3700 (53.9)	3169 (46.1)		1250 (61.7)	777 (38.3)	
Good	217 (50.8)	210 (49.2)		1463 (48.3)	1564 (51.7)		534 (56.7)	408 (43.3)	
Community-level factors									
Community employment			0.034*			0.170			0.316
Low	279 (54.9)	229 (45.1)		1943 (52.4)	1762 (47.6)		548 (57.7)	401 (42.3)	
Middle	237 (48.2)	255 (51.8)		1721 (51.5)	1619 (48.5)		698 (62.5)	418 (37.5)	
High	367 (57.9)	267 (42.1)		2287 (55.1)	1865 (44.9)		763 (61.8)	471 (38.2)	
Community education			0.356			0.007**			0.130
Low	363 (56.5)	279 (43.5)		2287 (56.1)	1793 (43.9)		672 (62.3)	407 (37.7)	
Middle	303 (51.3)	288 (48.7)		2045 (53.0)	1815 (47.0)		763 (63.0)	449 (37.0)	
High	217 (54.1)	184 (45.9)		1619 (49.7)	1638 (50.3)		574 (51.8)	434 (48.2)	
Community wealth			0.014*			< 0.001***			0.088
Low	389 (57.9)	283 (42.1)		2426 (56.2)	1894 (43.8)		695 (64.7)	379 (35.3)	
Middle	301 (55.2)	244 (44.8)		2141 (53.8)	1837 (46.2)		638 (60.7)	413 (39.3)	
High	193 (46.3)	224 (53.7)		1384 (47.7)	1515 (52.3)		676 (57.6)	498 (42.4)	
Community distance to HF			0.402			0.009**			0.128
Low	255 (54.0)	217 (46.0)		1367 (49.0)	1424 (51.0)		675 (60.9)	434 (39.1)	
Middle	315 (53.8)	271 (46.2)		2288 (53.8)	1967 (46.2)		798 (63.1)	466 (36.9)	
High	343 (56.6)	263 (43.4)		2296 (55.3)	1854 (44.7)		636 (62.0)	390 (38.0)	
Community ITN coverage			< 0.001***			< 0.001***			< 0.001***
Low	422 (61.6)	263 (38.4)		2803 (63.1)	1638 (36.9)		969 (72.8)	362 (27.2)	
Middle	314 (54.2)	265 (45.8)		2189 (52.4)	1988 (47.6)		697 (60.2)	460 (39.8)	
High	147 (39.7)	223 (60.3)		959 (37.2)	1620 (62.8)		343 (42.3)	468 (57.7)	
Community women’s autonomy in HC decisions			0.124			0.106			0.883
Low	357 (57.9)	260 (42.9)		2108 (55.1)	1719 (44.9)		611 (61.8)	378 (38.2)	
Middle	262 (50.1)	261 (49.9)		2033 (53.4)	1776 (46.6)		694 (60.8)	447 (39.2)	
High	263 (53.3)	230 (46.7)		1810 (50.8)	1751 (49.2)		704 (60.2)	465 (39.8)	
Community IRS coverage			0.989			0.521			0.736
Low	288 (36.5)	500 (63.5)		4262 (53.5)	3709 (46.5)		1490 (61.1)	947 (39.9)	
High	295 (54.0)	251 (46.0)		1689 (52.3)	1537 (47.7)		519 (60.2)	343 (39.8)	

*HF* health facility, *IRS* indoor residual spraying, *HH* household, *HC* health care, *ITN* insecticide-treated nets

* *p *< 0.05; ** *p *< 0.01; *** *p *< 0.001

^a^Pearson’s Chi square test

### Modelling approaches (measures of association)

#### Pregnant women

Table 2 shows the results of multilevel logistic regression analyses for the pregnant women group. Model III, which considers both individual- and community-level factors, shows that women who were from the central region, from urban areas, were secundigravida and multigravida, were from households with < 5 household members, were from households with ≥ 2 ITNs, and who did not perceive distance to health facility as a problem were more likely to use ITNs compared with their defined counterparts (aOR = 1.66, 95% CI 1.12–2.45; aOR = 1.63, 95% CI 1.04–2.55; aOR = 1.79, 95% CI 1.23–2.62; aOR = 1.97, 95% CI 1.28–3.28; aOR = 2.33, 95% CI 1.74–3.11; aOR = 4.25, 95% CI 3.19–5.64, and aOR = 1.44, 95% CI 1.09–1.89 respectively). Those living in high-ITN-coverage communities were more likely to use ITNs than those living in communities with low ITN coverage (aOR = 1.47, 95% CI 1.05–2.06).

**Table 2:** Multilevel analysis of factors associated with ITN use among pregnant women

Variable	Null model	Model I aOR (95% CI)	Model II aOR (95% CI)	Model III aOR (95% CI)
Individual-level factors				
Age (years)				
15–24		1.00		1.00
25–34		0.76 (0.54–1.08)		0.74 (0.53–1.05)
≥ 35		0.90 (0.55–1.49)		0.89 (0.54–1.47)
Education				
No education		1.00		1.00
Primary education		1.99 (0.78–1.83)		1.24 (0.81–1.92)
Secondary+		1.42 (0.86–2.36)		1.49 (0.89–1.49)
Occupation				
Unemployed		1.00		1.00
Employed		1.06 (0.83–1.35)		1.05 (0.81–1.37)
Religion				
Muslim and others		1.00		1.00
Catholic		0.86 (0.57–1.30)		0.82 (0.53–1.25)
Protestant		0.81 (0.57–1.15)		0.77 (0.54–1.11)
Wealth				
Poor		1.00		1.00
Middle		1.23 (0.89–2.61)		1.16 (0.85–1.57)
Rich		0.93 (0.68–1.27)		0.96 (0.61–1.51)
Region				
Northern		1.00		1.00
Central		1.82** (1.28–2.59)		1.66* (1.12–2.45)
Southern		1.27 (0.99–1.78)		1.15 (0.79–1.68)
Residence				
Rural		1.00		1.00
Urban		1.48* (1.02–2.14)		1.63* (1.04–2.55)
Altitude (m)				
< 1600		1.00		1.00
≥ 1600		0.38 (0.91–1.64)		0.33 (0.08–1.38)
Parity				
Primigravida		1.00		1.00
Secundigravida		1.79** (1.22–2.61)		1.79** (1.23–2.62)
Multigravida		1.92** (1.25–2.97)		1.97** (1.28–3.28)
Number of children under 5 years in HH				
One		1.00		1.00
None		0.99 (0.73–1.34)		1.01 (0.75–1.37)
≥ 2		1.19 (0.83–1.72)		1.19 (0.83–1.72)
Number of HH members				
≥ 5		1.00		1.00
< 5		2.38*** (1.78–3.18)		2.33*** (1.74–3.11)
Number of ITNs in HH				
< 2		1.00		1.00
≥ 2		4.68*** (3.57–6.15)		4.25*** (3.19–5.64)
IRS				
No		1.00		1.00
Yes		1.33 (0.84–2.10)		1.26 (0.77–0.26)
Media exposure				
No		1.00		1.00
Yes		1.05 (0.82–1.35)		1.06 (0.83–1.36)
Distance to HF				
Problem		1.00		1.00
No problem		1.39** (1.09–1.79)		1.44* (1.09–1.89)
Women’s autonomy in HC decisions				
Self		1.00		1.00
Husband alone		1.19 (0.78–1.83)		1.07 (0.74–1.54)
With others		0.91 (0.64–1.29)		0.92 (0.64–1.31)
Health behaviour				
Poor		1.00		1.00
Moderate		0.99 (0.69–1.44)		1.01 (0.69–1.45)
Good		1.34 (0.89–2.01)		1.35 (0.89–2.03)
Community-level factors				
Community employment				
Low			1.00	1.00
Middle			1.11 (0.85–1.48)	1.20 (0.88–1.64)
High			1.00 (0.75–1.33)	1.02 (0.72–1.44)
Community education				
Low			1.00	1.00
Middle			1.06 (0.81–1.40)	1.04 (0.76–1.41)
High			0.70* (0.51–0.97)	0.75 (0.51–1.09)
Community wealth				
Low			1.00	1.00
Middle			1.12 (0.84–1.47)	1.16 (0.85–1.57)
High			1.21 (0.85–1.71)	0.96 (0.61–1.51)
Community distance to HF				
Low			1.00	1.00
Middle			0.87 (0.64–1.18)	1.04 (0.73–1.47)
High			0.78 (0.57–1.08)	1.03 (0.69–1.54)
Community ITN coverage				
Low			1.00	1.00
Middle			1.44** (1.09–1.91)	1.16 (0.76–1.41)
High			2.33*** (1.74–3.12)	1.47* (1.05–2.06)
Community women’s autonomy in HC decisions				
Low			1.00	1.00
Middle			1.22 (0.92–1.59)	1.16 (0.73–1.47)
High			1.09 (0.83–1.45)	1.03 (0.73–1.38)
Community IRS coverage				
Low			1.00	1.00
High			1.03 (0.81–1.32)	1.02 (0.76–1.35)
Measure of variation				
Area variance (95% CI)	0.429 (0.202–0.912)	0.321 (0.117–0.881)	0.231 (0.068–0.785)	0.259 (0.077–0.873)
ICC (%)	11.6	8.9	6.6	7.3
MOR	1.87	1.71	1.58	1.62
PCV (%)	Ref.	25.2	46.2	39.6
Model fit statistic				
AIC	2220.7	2024.2	2192.2	2036.9

Null model contains no explanatory variables; Model I includes individual-level factors only; Model II includes community-level factors only; Model III includes both individual-level and community-level factors

*ITN* insecticide-treated nets, *HF* health facility, *HH* household, *IRS* indoor residual spraying, *HC* health care, *ICC* intraclass correlation coefficient, *MOR* median odds ratio, *PVC* proportional change in variance, *AIC* Akaike information criterion

* *p *< 0.05, ** *p *< 0.01, *** *p *< 0.001

#### Non-pregnant women with children under 5 years

Among non-pregnant women with children under 5 years (Table 3), Model III showed that women from middle-wealth households, from the central and southern regions, from urban areas, who were secundigravida and multigravida, who had ≥ 2 children under 5 years, from households with < 5 members, from households with ≥ 2 ITNs, and who had good health behaviour programme were significantly more likely to use ITNs than their defined counterparts. Those from communities with middle and high ITN coverage and with high women’s autonomy in health care decisions were more likely to use ITNs than women from communities with low ITN coverage and low women’s autonomy in health care decisions (aOR = 1.19, 95% CI 1.02–1.38; aOR = 1.35, 95% CI 1.14–1.60; and aOR = 1.18, 95% CI 1.00–1.38, respectively).

**Table 3:** Multilevel Analysis of factors associated with ITN use among nonpregnant women with children under 5 years

Variable	Null model	Model I aOR (95% CI)	Model II aOR (95% CI)	Model III aOR (95% CI)
Individual-level factors				
Age (years)				
15–24		1.00		1.00
25–34		0.95 (0.83–1.08)		0.95 (0.84–1.09)
≥ 35		0.87 (0.75–1.02)		0.88 (0.75–1.02)
Education				
No education		1.00		1.00
Primary education		1.12 (0.97–1.29)		1.03 (0.90–1.17)
Secondary+		1.44*** (01.20–1.73)		1.04 (0.92–1.76)
Occupation				
Unemployed		1.00		1.00
Employed		0.95 (0.85–1.04)		0.94 (0.85–1.05)
Religion				
Muslim and others		1.00		1.00
Catholic		0.98 (0.81–1.17)		0.93 (0.78–1.12)
Protestant		0.94 (0.80–1.10)		0.90 (0.77–1.06)
Wealth				
Poor		1.00		1.00
Middle		1.13* (1.00–1.28)		1.13* (1.01–1.28)
Rich		1.04 (0.92–1.17)		1.06 (0.93–1.20)
Region				
Northern		1.00		1.00
Central		1.53*** (1.28–1.83)		1.46*** (1.19–1.78)
Southern		1.49*** (1.25–1.77)		1.40*** (1.16–1.70)
Residence				
Rural		1.00		1.00
Urban		1.42* (1.19–1.69)		1.48** (1.18–1.84)
Altitude (m)				
< 1600		1.00		1.00
≥ 1600		0.69 (0.29–1.69)		0.63 (0.25–1.52)
Parity				
Primigravida		1.00		1.00
Secundigravida		5.11*** (2.69–9.69)		4.97*** (2.62–9.43)
Multigravida		4.67*** (2.47–8.83)		4.55*** (2.40–8.61)
Number of children under 5 years in HH				
One		1.00		1.00
≥ 2		1.12* (1.01–1.23)		1.12* (1.01–1.24)
Number of HH members				
≥ 5		1.00		1.00
< 5		1.85*** (1.65–2.07)		1.85*** (1.65–2.08)
Number of ITNs in HH				
< 2		1.00		1.00
≥ 2		7.22*** (6.52–7.99)		6.89*** (6.19–7.65)
IRS				
No		1.00		1.00
Yes		1.09 (0.89–1.33)		1.06 (0.86–1.31)
Media exposure				
No		1.00		1.00
Yes		1.07 (0.97–1.77)		1.06 (0.96–1.17)
Distance to HF				
Problem		1.00		1.00
No problem		0.98 (0.89–1.08)		0.98 (0.88–1.09)
Women’s autonomy in HC decisions				
Self		1.00		1.00
Husband alone		1.03 (0.91–1.18)		1.02 (0.90–1.17)
With others		0.06 (0.94–1.20)		1.04 (0.92–1.18)
Health behaviour				
Poor		1.00		1.00
Moderate		1.00 (0.85–1.15)		1.00 (0.87–1.16)
Good		1.19* (1.01–1.39)		1.19* (1.01–1.39)
Community-level factors				
Community employment				
Low			1.00	1.00
Middle			1.09 (0.96–1.25)	1.08 (0.92–1.25)
High			1.01 (0.87–1.16)	0.97 (0.82–1.14)
Community education				
Low			1.00	1.00
Middle			1.04 (0.90–1.19)	0.96 (0.82–1.12)
High			0.95 (0.81–1.11)	0.94 (0.78–1.14)
Community wealth				
Low			1.00	1.00
Middle			1.00 (0.87–1.15)	0.99 (0.85–1.16)
High			1.05 (0.88–1.25)	0.81 (0.64–1.01)
Community distance HF				
Low			1.00	1.00
Middle			0.89 (0.77–1.04)	0.92 (0.77–1.09)
High			0.90 (0.77–1.06)	0.97 (0.79–1.17)
Community ITN coverage				
Low			1.00	1.00
Middle			1.67*** (1.46–1.93)	1.19* (1.02–1.38)
High			3.05*** (2.64–3.52)	1.35*** (1.14–1.60)
Community women’s autonomy in HC decisions				
Low			1.00	1.00
Middle			1.08 (0.94–1.23)	1.01 (0.86–1.17)
High			1.27* (1.11–1.45)	1.18* (1.00–1.38)
Community IRS coverage				
Low			1.00	1.00
High			1.05 (0.94–1.23)	1.03 (0.89–1.19)
Measure of variation				
Area variance (95% CI)	0.535 (0.449–0.637)	0.403 (0.325–0.498)	0.297 (0.237–0.372)	0.380 (0.305–0.473)
ICC (%)	13.9	10.9	8.3	10.4
MOR	2.01	1.83	1.68	1.80
PCV (%)	Ref.	24.7	44.5	29.0
Model fit statistic				
AIC	14,990.2	13,012.8	14,753.5	13,017.2

Null model contains no explanatory variables; Model I includes individual-level factors only; Model II includes community-level factors only; Model III include both individual-level and community-level factors

*ITN* insecticide treated nets, *HF* health facility, *HH* household, *IRS* indoor residual spraying, *HC* health care, *ICC* intraclass correlation coefficient, *MOR* median odds ratio, *PVC* proportional change in variance, *AIC* Akaike’s information criterion

* *p *< 0.05, ** *p *< 0.01, *** *p *< 0.001

#### Non-pregnant women without children under 5 years

For non-pregnant women without children under 5 years (Table 4), Model III revealed that only those from the central and southern regions, from rural areas, from households with < 5 members, and from households with ≥ 2 ITNs were more likely to use ITNs than their defined counterparts. However, Catholic women were less likely to use ITNs than Muslims (aOR = 0.64, 95% CI 0.44–0.91). Women living in the communities with middle and high ITN coverage were significantly more likely to use ITNs than those living in the communities with low ITN coverage.

**Table 4:** Multilevel analysis of factors associated with ITN use among nonpregnant women without children under 5 years

Variable	Null model	Model I aOR (95% CI)	Model II aOR (95% CI)	Model III aOR (95% CI)
Individual-level factors				
Age (years)				
15–24		1.00		1.00
25–34		1.08 (0.75–1.56)		1.05 (0.77–1.59)
≥ 35		1.03 (0.71–1.50)		1.07 (0.74–1.55)
Education				
No education		1.00		1.00
Primary education		0.95 (0.74–1.22)		0.98 (0.76–1.28)
Secondary+		0.89 (0.65–1.24)		0.94 (0.67–1.32)
Occupation				
Unemployed		1.00		1.00
Employed		1.01 (0.82–1.24)		1.02 (0.82–1.28)
Religion				
Muslim and others		1.00		1.00
Catholic		0.63* (0.44–0.90)		0.64* (0.44–0.91)
Protestant		0.76 (0.55–1.05)		0.75 (0.54–1.03)
Wealth				
Poor		1.00		1.00
Middle		0.92 (0.69–1.19)		0.93 (0.69–1.99)
Rich		0.78 (0.54–1.35)		1.81 (0.56–1.17)
Region				
Northern		1.00		1.00
Central		1.53** (1.15–2.03)		1.53** (1.12–2.09)
Southern		1.31* (1.00–1.72)		1.34* (1.00–1.81)
Residence				
Rural		1.00		1.00
Urban		1.53** (1.18–1.98)		1.57** (1.14–2.16)
Altitude (m)				
< 1600		1.00		1.00
≥ 1600		0.68 (0.17–2.82)		0.51 (0.54–1.04)
Parity				
Primigravida		1.00		1.00
Secundigravida		0.91 (0.69–1.19)		0.93 (0.63–1.28)
Multigravida		0.78 (0.54–1.35)		0.81 (0.56–1.17)
Number of HH members				
≥ 5		1.00		1.00
< 5		1.67*** (1.37–2.03)		1.66*** (1.37–2.02)
Number of ITNs in HH				
< 2		1.00		1.00
≥ 2		11.89*** (9.6–14.7)		10.72*** (8.59–13.37)
IRS				
No		1.00		1.00
Yes		1.45 (0.99–2.15)		1.24 (0.82–1.88)
Media exposure				
No		1.00		1.00
Yes		1.10 (0.91–1.34)		1.11 (0.91–1.35)
Distance to HF				
Problem		1.00		1.00
No problem		1.01 (0.84–1.22)		1.09 (0.89–1.35)
Women’s autonomy in HC decisions				
Self		1.00		1.00
Husband alone		0.99 (0.77–1.29)		0.99 (0.97–1.28)
With others		1.14 (0.91–1.43)		1.11 (0.87–1.40)
Health behaviour				
Poor		1.00		1.00
Moderate		1.21 (0.89–1.65)		1.21 (0.89–1.65)
Good		1.22 (0.87–1.70)		1.22 (0.88–1.71)
Community-level factors				
Community employment				
Low			1.00	1.00
Middle			1.02 (0.82–1.26)	0.97 (0.76–1.24)
High			1.02 (0.82–1.28)	0.95 (0.73–1.34)
Community education				
Low			1.00	1.00
Middle			0.85 (0.68–1.06)	0.94 (0.73–1.20)
High			0.79 (0.61–1.01)	0.93 (0.69–1.26)
Community wealth				
Low			1.00	1.00
Middle			1.13 (0.89–1.42)	1.05 (0.81–1.36)
High			1.23 (0.93–1.61)	0.99 (0.69–1.41)
Community distance to HF				
Low			1.00	1.00
Middle			0.84 (0.67–1.06)	1.14 (0.86–1.49)
High			0.96 (0.75–1.25)	1.36 (0.99–1.86)
Community ITN coverage				
Low			1.00	1.00
Middle			2.00*** (1.59–2.50)	1.36* (1.06–1.75)
High			3.95*** (3.14–4.98)	1.49** (1.15–1.96)
Community women’s autonomy in HC decisions				
Low			1.00	1.00
Middle			1.03 (0.68–1.05)	0.94 (0.73–1.21)
High			1.19 (0.97–1.49)	1.12 (0.87–1.45)
Community IRS coverage				
Low			1.00	1.00
High			1.16 (0.96–1.42)	1.22 (0.97–1.54)
Measure of variation				
Area variance (95% CI)	0.684 (0.492–0.949)	0.347 (0.191–0.631)	0.293 (0.168–0.511)	0.293 (0.148–0.579)
ICC (%)	17.2	9.6	8.2	8.2
MOR	2.20	1.76	1.68	1.68
PCV (%)	Ref.	49.3	57.2	57.2
Model fit statistic				
AIC	4279.9	3496.8	4135.6	3504.4

Null model contains no explanatory variables; Model I includes individual-level factors only; Model II includes community-level factors only; Model III include both individual-level and community-level factors

*ITN* insecticide treated nets, *HF* health facility, *HH* household, *IRS* indoor residual spraying, *HC* health care, *ICC*  intraclass correlation coefficient, *MOR* median odds ratio, *PVC* proportional change in variance, *AIC* Akaike’s information criterion

* *p *< 0.05, ** *p *< 0.01, *** *p *< 0.001

### Measures of variation (random effects)

Table 2 also shows the results of measures of variation. In the null model, significant variation in ITN use among pregnant women across communities (σ^2^ = 0.429, 95% CI 0.202–0.912) was observed, justifying the use of multilevel analysis for modelling. The ICC was 11.6%, suggesting that the variation in ITN use may be attributable to unobserved community characteristics. In comparison with the null model, the variation shown for ITN use from Model I to Model III remained significant across communities. From the null model to Model III, ICC between communities dropped slightly, suggesting that controlling for individual- and community-level factors slightly reduced the proportion of variance in ITN use between communities. In the final model, the MOR showing the effects of community heterogeneity was 1.62, and 39.6% of the variance in the odds of using ITN across communities was explained by both individual- and community-level factors, as indicated by the PCV.

Similarly, in Tables 3 and 4, Model III shows that variance in ITN use was significant even after controlling for individual- and community-level factors in both groups of non-pregnant women. The community effects on ITN use were higher among non-pregnant women with children under 5 years (MOR = 1.80) than among pregnant and non-pregnant women without children under 5 years (MOR = 1.62 and 1.68, respectively).

## Discussion

This study demonstrated that both individual- and community-level factors influence ITN usage among WOCBA in Malawi. Furthermore, it revealed that factors affecting ITN utilization may vary across different groups of women and that prevention and intervention programmes should be group-specific. Selected findings are that non-pregnant women with children under 5 years living in communities where women had high levels of autonomy in health care decisions had an 18% greater odds of using ITNs than those from communities where such women who had low levels of autonomy in health care decisions. Furthermore, pregnant women who did not perceive the distance to their nearest health facility to be a problem were 1.44 times more likely to use ITNs than those who perceived it as a problem. In addition, regional variation in ITN use was observed across all groups of women.

ITN usage was similar between pregnant women and non-pregnant women with children under 5 years (45.9% and 46.9%, respectively), whereas it was lower (39.1%) among non-pregnant women without children under 5 years. This difference may be accounted for by the free ITNs received during ANC and EPI visits by pregnant women and non-pregnant women with children under 5 years [11, 34, 35]. In Malawi, a national-wide ITN distribution campaign was implemented in 2012 [10]. However, during the 2014–2015 national ITN distribution campaign, only six districts were covered, while implementation of the programme in the other districts was postponed [24]. This could moderately explain the observed low ITN usage across all women groups in Malawi in the 2015–2016 MDHS dataset. This study’s result is similar to the low ITN usage rate reported for WOCBA in sub-Saharan Africa [11, 36]. As inadequate rates of ITN use among the vulnerable population were observed, issues related to ITN use are critical public health concerns. It is imperative to propose and implement approaches to increasing coverage, such as more frequent campaigns or additional distribution systems to maintain coverage between campaigns.

Across all groups, women from communities with high ITN coverage were more likely to use an ITN than those from communities with low ITN coverage (1.47-, 1.35-, and 1.49-fold increased odds of ITN use among pregnant women, non-pregnant women with children under 5 years, and non-pregnant women without children under 5 years, respectively). Studies have shown that access to ITN is a key determinant for ITN use [37, 38]. Therefore, this study underscores the importance of achieving universal ITN access/coverage, which might ultimately have an influence on ITN utilization [24].

Empowerment of women has been found to influence several health outcomes and maternal health service utilization [19, 20, 39, 40]. This study reports that non-pregnant women with children under 5 years who lived in communities where women have high levels of autonomy in health care decisions had 1.18-fold greater odds of using an ITN than those living in communities where women have low levels of this autonomy. In communities where the percentage of women empowered to make health care decisions is high, women may be more likely to use medical services, such as those providing ITNs, and are more likely to be empowered with regards to other factors like education [13, 41, 42]. The health care empowerment factor was not significant in the groups of pregnant women and non-pregnant women without children under 5 years. Pregnant women are likely to be younger than non-pregnant women with children under 5 years, and studies have shown that younger women are less likely to be empowered than older women [43]. Non-pregnant women without children under 5 years may have less awareness of the importance of ITNs in malaria prevention because they have not attended ANC or EPI appointments, which include discussion of critical information about malaria [44]. These results highlight the benefits that result from empowering women.

Ultimately, the community-level results suggested that women living in the same community may be exposed to common influences that correlate with ITN usage. As shown in the multilevel analyses, even after controlling for individual- and community-level factors, the variation in ITN use remained significant with MOR ≥ 1.6, suggesting that communities have significant effects on ITN use among WOCBA in Malawi. Thus, policy makers and programme designers should consider community characteristics when designing ITN programmes to ensure their effectiveness.

The individual-level factors showed that women from the central and southern regions, from urban areas, from households with few members, and from households with ≥ 2 ITNs were more likely to use ITNs than their counterparts across all three groups. This finding aligns with results reported for several countries [11, 24, 37, 45–47]. A Malawian study reported district variation in ITN usage among WOCBA; the present study investigated further and revealed regional variation in ITN usage among WOCBA groups [11]. Malaria parasitaemia was reported to be more prevalent in the central (50%) and southern (42%) regions compared with the northern (23%) region of Malawi [47]. Women from the central and southern regions were thus found to be more likely to use ITNs than women from the northern region because malaria-prevention programmes tend to focus more on the areas where malaria is more endemic, resulting in higher ITN usage in these areas. In urban areas, women may have access to superior malaria-prevention resources, such as repellants and ITNs. Additionally, they may have more knowledge about the importance of malaria prevention because of more extensive media exposure and relatively high educational attainment; thus these women are more likely to use ITNs than women living in rural areas [13]. These findings are consistent with findings in other countries where high levels of ITN use among urban women were reported [45]. Households with more members may have high person: TN ratios, resulting in lower likelihoods that the women of the households would use the ITNs [24, 48]. Greater ITN availability per household may increase ITN usage among women living in large households [37, 46]. Furthermore, greater numbers of ITNs per household may lead to increased knowledge about the importance of ITNs in malaria prevention among individuals living within the households.

Consistent with the results of a Ugandan study, the current study showed that parity was associated with ITN use among pregnant and non-pregnant women with children under 5 years, with secundigravida and multigravida women being more likely to use ITNs than primigravida women [14]. In addition to having received free ITNs through previous medical care, secundigravida and multigravida women may have been more exposed to information about malaria through previous ANC and EPI appointments [44].

Among the pregnant women, those who did not perceive distance to the nearest health care facility to be a problem were more likely to use ITNs. Similar findings were reported from Mali, where ITN ownership was influenced by distance to health facility [48]. Apart from influencing ITN ownership, health facilities may play a role in promoting its use as was observed in an earlier Malawian study [49]. Improving health facility accessibility is thus essential; when women are able to attend clinics for ANC they may receive free ITNs and be exposed to critical information about malaria. Government efforts should aim to improve the accessibility of health services, especially for pregnant women.

Among the non-pregnant women with children under 5 years, analysis from the present study showed that those with good health behaviours and from middle-wealth households were more likely to use ITNs than those with poor health behaviours and from poor households. In Nigeria, middle and high wealth levels were associated with women’s ITN usage [18]. By contrast, a Malawian study found no association between wealth and ITN usage [11]. The association in this study was observed in non-pregnant women only, suggesting that free ITN distribution at ANC appointments may eliminate the wealth-related disparity in ITN usage [11]. The results of the present study suggest that non-pregnant women with low socioeconomic status who have children under 5 years should be prioritized during mass ITN distribution. In addition, the results suggest that specific health behaviours, such as ITN use, are part of an interdependent system of good health practices, especially for women caring for young children.

Among the non-pregnant women without children under 5 years, Catholic women were less likely to use ITNs than Muslim women. This finding is similar to that of a study performed in Kenya where Christians were found to be less likely to use ITNs than Muslims [16]. The reasons for these observed differences are still unknown; however, religious beliefs have been shown to influence health care utilization in many countries [50].

This study used a nationally representative data sample; hence, the results may be generalized for Malawian women and specifically for various WOCBA subgroups. In addition, this study examined a wide range of individual- and community-levels factors influencing ITN use. However, due to the use of administratively defined boundaries as proxies for communities, non-differential misclassification of women into unfitted administrative communities may have yielded information biases, which would have compromised the findings. Although perceived distance to health facility may affect health behaviours in addition to actual distance, the use of perceived distance to health facility only in this study is prone to perception bias. And as such, other studies need to consider collecting information of the actual distance to health facility. In addition, knowledge and perception of ITNs were not included in the analyses because the MDHS did not gather this data. However, media exposure was included and may serve as a proxy for knowledge. Although statistically the low VIF values did not warrant concerns on multicollinearity problems, due to the complexity of human behaviour, the potential correlations between factors that may affect ITN use should still be cautiously considered in the interpretations and implications of the study results. Lastly, the present study was cross-sectional; therefore, cause–effect relationships could not be inferred.

## Conclusion

This study discovered that both individual- and community-level factors influence ITN usage among women in Malawi. Regional variation in ITN use in Malawi was observed; therefore, prevention efforts should focus on increasing ITN usage among women in the northern region to achieve the World Health Organization global goal of malaria elimination by 2030. Furthermore, public health interventions should target women with certain individual- and community-level characteristics that are deemed significant among the various WOCBA groups. Future research should investigate whether mothers’ ITN usage is associated with ITN use among children under 5 years, because children are a vulnerable population of concern in malaria-prevention efforts.

## Abbreviations

ITN: insecticide-treated nets
MIP: malaria in pregnancy
ANC: antenatal care
WOCBA: women of childbearing age
aOR: adjusted odds ratio
MOR: median odds ratio
HF: health facility
HH: household
IRS: indoor residual spraying
HC: health care
ICC: intraclass correlation coefficient
PVC: proportional change in variance
AIC: Akaike information criterion
EPI: expanded programme on immunization
MDHS: Malawi Demographic Health Survey
SEAs: standard enumeration areas
ICF: International Classification of Functioning Disability and Health
